# Effectiveness of a combined online and in-person training model in improving village health workers’ counselling quality and infant and young child feeding practices: a mixed-methods study in rural China

**DOI:** 10.7189/jogh.16.04177

**Published:** 2026-06-19

**Authors:** Qiong Wu, Xinya Zhu, Xiaotong Wang, Na Meng, Jinbo Li, Ailing Peng, Yanfeng Zhang, Suying Chang

**Affiliations:** 1 Department of Integrated Early Childhood Development, Capital Center for Children’s Health, Capital Medical University, Capital Institute of Pediatrics, Beijing, China; 2 Child Health and Development Section, UNICEF China, Beijing, China; 3 Department of Pediatrics, West China Second University Hospital, Sichuan University, Chengdu, China; 4 Changyang County Maternal and Child Health Hospital, Hubei, China

## Abstract

**Background:**

To promote optimal nutrition and appropriate infant and young child feeding (IYCF), a national programme in China implemented a combined two-day online and one-day in-person IYCF counselling training (‘2 + 1’ training model) for primary health workers. However, evidence on its effectiveness remains limited. We aimed to assess improvements in the quality of counselling by village health workers (VHWs) and changes in key IYCF practices.

**Methods:**

We conducted a mixed-methods study in Changyang County, Hubei Province, China, from September 2023 to August 2025. Townships/villages were allocated into intervention and control groups. Intervention VHWs received the ‘2 + 1’ training model and provided IYCF counselling. We assessed counselling quality through non-participant observation at baseline and endline, with the quality rate defined as achieving at least four of six key skills. IYCF practices were evaluated through two cross-sectional surveys of caregivers of children aged six to 23 months, administered via a WeChat online questionnaire. In addition, we conducted focus group discussions and semi-structured interviews.

**Results:**

At baseline, the counselling quality rate was low in both groups (12.0% *vs*. 3.9%, *P* = 0.0072). At endline, it increased substantially in the intervention group (84.8% *vs*. 32.2% in controls). The overall counselling skills score showed similar trends. VHWs reported that their IYCF knowledge, skills and communication confidence improved after training. For the IYCF practices, there was no difference in minimum dietary diversity (MDD), minimum meal frequency, and minimum acceptable diet (MAD) between the two groups at baseline; however, at endline, the intervention group achieved higher MDD (70.2% *vs*. 59.4%, *P* = 0.0415) and MAD (58.4% *vs*. 46.7%, *P* = 0.0342) prevalence compared to the control group. Caregivers expressed high satisfaction with the counselling services.

**Conclusions:**

The combined online and in-person IYCF counselling training could effectively improve VHWs’ feeding counselling quality and subsequently maintain IYCF practices in a rural area in China.

Appropriate infant and young child feeding (IYCF) is fundamental for optimal nutrition and development during the first two years of life, with lasting effects on health throughout childhood and adulthood [1]. Nevertheless, inadequate feeding remains widespread globally: only 45.7% of infants under six months are exclusively breastfed, and approximately one-third of children aged six to 23 months meet the minimum dietary diversity (MDD) required for healthy growth [2,3]. Similarly, IYCF practices in rural China are suboptimal. It is reported that among children aged six to 23 months in rural areas, the prevalence of MDD, minimum meal frequency (MMF), and minimum acceptable diet (MAD) is 68.9%, 77.9%, and 46.4%, respectively [4]. Given the rapid growth, high nutritional demands, and heightened vulnerability to illness among infants and young children aged <2 years, improving feeding practices during this period remains a public health priority [5].

IYCF counselling, a type of support provided to parents or other caregivers, has been shown to increase caregivers’ knowledge and improve breastfeeding, complementary feeding, and child growth outcomes [6–8]. To promote child feeding and nutrition in China, the National Health Commission (NHC) and the United Nations Children’s Fund (UNICEF) China jointly developed and released the ‘Infant and Young Child Feeding Counselling: A Training Curriculum and Practical Guide for Primary Health Workers’ in Chinese in 2021 [9]. To expand access to training nationwide, a combined online and in-person model was subsequently introduced for primary health workers: a two-day online video-based IYCF counselling course followed by a one-day in-person session for practical skill application – the ‘2 + 1’ training model. This model was piloted in five counties of Qinghai Province between September 2021 and February 2022, and was found to be acceptable, feasible, low-cost, and scalable across rural China [10].

Based on these pilot findings, the NHC launched the ‘National Training Program for Improving Counselling Capacity on Infant and Young Child Nutrition and Feeding’ in May 2022, extending the ‘2 + 1’ training model to 1212 counties across 31 provinces. To further strengthen service delivery, the NHC also issued the ‘Guidelines for Infant and Young Child Nutrition and Feeding Assessment Services’ [11]. Despite nationwide rollout, the effectiveness of the ‘2 + 1’ training model in improving primary health workers’ counselling skills and IYCF practices in China has not yet been systematically evaluated. In this study, we aimed to assess the effectiveness of the ‘2 + 1’ training model in improving the quality of IYCF counselling by village health workers (VHWs) and service performance, and to examine its subsequent impact on IYCF practices in rural China. We hypothesised that the implementation of the ‘2 + 1’ training model would improve the IYCF counselling skills of village health workers, enhance the quality of counselling delivered, and subsequently improve population-based IYCF practices.

## METHODS

### Study design

We applied a mixed-method approach in Changyang County, Hubei Province, China, between September 2023 and August 2025. The quantitative component was conducted as a pragmatic community-based intervention study embedded within the implementation of the national IYCF training programme. We aimed to assess improvements in the quality of IYCF counselling by VHWs and in population-based IYCF practices under real-world service delivery conditions.

We collected qualitative data through two focus group discussions with local health workers and six semi-structured interviews with caregivers to gain deeper insight into programme implementation and challenges. We used the qualitative component to provide contextual understanding of the intervention and to complement the quantitative findings [12]. We considered quantitative and qualitative results together to provide a more comprehensive understanding of the implementation of the ‘2 + 1’ training model. Results are presented first for quantitative data, followed by qualitative findings that help interpret them.

### Participants

VHWs and caregivers with children aged six to 23 months were participants in both quantitative and qualitative studies. For VHWs, we recruited all village-level maternal and child health (MCH) workers in both intervention and control townships who were village women’s directors and were responsible for folic acid and Ying Yang Bao distribution, and postpartum visits, *etc.* For caregivers, the inclusion criteria were as follows: children aged between six and 23 months; being a primary caregiver; having no plans to go out in the coming year; being able to read in Mandarin; using WeChat (Tencent Holdings Limited, Shenzhen, Guangdong, China); and having access to the internet. Exclusion criteria were: children with a structural or genetic birth defect, such as neural tube defects, congenital heart disease or any inherited metabolic diseases and caregivers who refused to participate. In addition, we recruited county- and township-level MCH workers responsible for training VHWs and for providing supervision to participate in focus groups in the qualitative study.

### Study setting

Hubei Province lies in central China, with an area of about 190 000 km^2^. By the end of 2023, the total population was 58.38 million, of which the rural population accounted for 34.53%. Hubei Province has 35 counties. Changyang County is located in the southwest part of Hubei Province. It has a total population of 319 100 (with 6265 under-five children), and the annual per capita net income of rural residents was CNY 15 874 (USD 2227.68) in 2023, which was lower than the national average (CNY 21 691 (USD 3044.01) [13]. Administratively, Changyang County consists of 11 townships and 160 villages. It is also one of the 1212 counties implementing the ‘National Training Program for Improving Counselling Capacity on Infant and Young Child Nutrition and Feeding.’

### Quantitative phase

We designed the quantitative phase of the study as a pragmatic community-based intervention study. In the intervention townships, VHWs received the ‘2 + 1’ training model for IYCF counselling, and then provided IYCF counselling services to caregivers of children aged six to 23 months, whereas in the control township, no interventions took place. First, we assessed the improvement in counselling quality among VHWs from September 2023 to June 2024. Subsequently, we evaluated IYCF practices in the two groups from December 2024 to August 2025 to assess the effectiveness of the interventions.

### Allocation of intervention and control areas

Considering the administrative structure of Changyang County, a two-step allocation approach was adopted to assign intervention and control areas. For the largest township, which contains 26 villages, villages were allocated in equal proportion to either the intervention or the control group using a random allocation procedure to maintain balance in the number of participating villages. For the remaining 10 townships, allocation was performed at the township level. Townships were first paired based on their total population and number of administrative villages to ensure comparability, and within each pair, one township was randomly assigned to the intervention group and the other to the control group. We adopted this allocation approach to ensure operational feasibility and balance in the number of intervention sites while implementing the training and supervision activities across the county.

### Intervention and delivery channels

For all VHWs in the intervention group, we implemented the ‘2 + 1’ training model for IYCF counselling from November to December 2023. VHWs were first required to complete a two-day online, video-based training course independently on their computers or smartphones. This course is freely available in Chinese on the official website. The video-based training adopts a scenario-based format, featuring one trainer and four trainees who demonstrate key IYCF knowledge and counselling skills. The previous paper presented a comprehensive description of the ‘2 + 1’ training model [10].

After all VHWs had completed the online training course and obtained a certificate of completion, one-day in-person practical training sessions were organised to enhance their practical skills in IYCF counselling. VHWs from two nearby townships were gathered and trained, and a total of four training sessions were conducted. Following the ‘2 + 1’ training, each trained village health worker was encouraged to integrate IYCF counselling services into their routine child care service provision for children in the intervention group. However, supervision during the early implementation period indicated that the delivery of counselling activities was poor due to insufficient programmatic support and a lack of incentives.

Following the endline counselling quality assessment in June 2024, the period from July to November 2024 was primarily devoted to strengthening counselling implementation, including planning activities and programme coordination. Following refresher training for VHWs conducted in December 2024, intensive counselling implementation and supervision were initiated. VHWs were required to provide at least three counselling sessions to each eligible caregiver in their own villages, with CNY 10 for each counselling session as the programme incentive. Meanwhile, MCH workers at the county and township levels conducted two rounds of supportive supervision – they observed VHWs’ counselling sessions without interference and provided targeted feedback after each session to reinforce their counselling skills.

**Figure 1:**
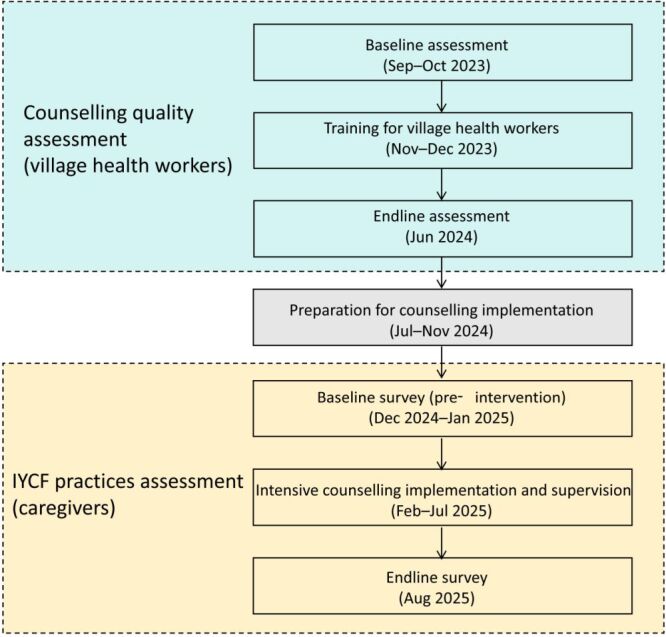
Schematic programme timeline.

In addition, we developed a health education leaflet tailored to caregivers of children aged six to 23 months in the intervention group. This leaflet included key information on IYCF, such as the importance of continued breastfeeding, minimum dietary diversity requirements, recommended minimum meal frequency, the importance of animal-source foods, complementary food texture and consistency by age groups, and responsive feeding. The leaflet was distributed by VHWs during IYCF counselling sessions for caregivers. From February to August 2025, intensive counselling implementation and supervision were conducted (**Figure 1**).

### Sample size and sampling

Due to limited data on the quality assessment of IYCF counselling in China, we assumed a baseline prevalence of 50% for high-quality IYCF counselling and expected a 17% increase in prevalence with the intervention. Using a significance level of 5% and a power of 80%, the required sample size was calculated to be 128 counselling services per group. Taking into account an estimated response loss of 20%, we aimed to collect 160 counselling sessions per group. Considering the daily number of children aged <2 years receiving health services and the logistical arrangements for these services, each group was required to survey 32 VHWs, and each health worker provided counselling services to five caregivers of children aged six to 23 months. Convenience sampling was employed to recruit caregivers who accessed counselling services.

The sample size for the evaluation of IYCF practices in this study was estimated using national data and Power Analysis & Sample Size, version 15.0 (NCSS, LLC, Kaysville, Utah, USA). With 46.4% of the MAD expected, a 17% improvement, 80% power, and a 5% significance level, we calculated that a sample size of 131 caregivers per group with children aged six to 23 months would be sufficient for all key indicators. We oversampled 20% of children to compensate for potential nonresponse and the relatively high mobility of children and caregivers in the study county. All eligible caregivers in both intervention and control groups were invited to participate in both surveys.

### Survey instrument

For the counselling quality assessment, we adapted the tool from the ‘Supportive supervision tool 4: Observation checklist for IYCF counselling’ in the World Health Organization (WHO) ‘Infant and young child feeding counselling: an integrated course: trainer’s guide, second edition’ [14]. This tool evaluated whether health workers performed six key counselling skills, including obtaining the correct infant age, assessing infant feeding, analysing feeding problems, providing actionable advice to caregivers, appropriately using the counselling card and communication skills during counselling sessions. Each key counselling skill was assigned a score of one point, resulting in a total of six points for the overall counselling skills assessment. We set up the assessment Checklist on the Sojump platform (Changsha Ranxing Information Technology Co., Ltd, Changsha, China) to create an electronic questionnaire.

The survey questionnaire for the IYCF practices evaluation was in line with our previous studies [15–17] and was used to collect data on children’s complementary feeding practices and caregivers’ feeding knowledge and information sources. We set up the questionnaire on the Sojump and obtained the quick response (QR) code for the electronic questionnaire.

### Data collection

We conducted baseline and endline counselling quality assessment in September–October 2023, and June 2024, respectively. Staff from the Capital Institute of Paediatrics in Beijing acted as supervisors. Township maternal and child health workers were trained as interviewers to collect assessment data using smartphones. Before data collection, all interviewers received standardised training on the observation procedures and scoring criteria based on the UNICEF IYCF counselling training modules to ensure consistency in data collection. During data collection, a non-participant observation method was used. Blinding of observers to the intervention status was not feasible because the interviewers were township maternal and child health workers who routinely supervised village health workers in the study areas. After obtaining both oral and written informed consent from the VHWs and caregivers, interviewers conducted observations from a position one to 1.5 m to the side of the counselling scene. Throughout the process, they used the electronic assessment checklist to document the IYCF counselling skills of VHWs in real time. The checklist included predefined criteria for each counselling skill domain to promote standardised assessment and reduce observers’ subjective judgement. The observation strictly adhered to the ‘principle of minimal intervention’: no verbal or non-verbal interaction was initiated with health workers or caregivers; no interference was made in the counselling process. The interviewers were also required to record the start time and end time of each counselling.

To evaluate IYCF practices, we conducted repeated cross-sectional surveys: a baseline survey from December 2024 to January 2025 and an endline survey in August 2025. The questionnaire QR code was distributed to caregivers via the three-tier health care system (county, township, village). The distribution process followed a hierarchical pathway. First, we sent the QR code to the county-level coordinator, who then forwarded it to the township-level coordinators and VHWs. VHWs provided study explanations to caregivers, shared the QR codes via WeChat, and assisted caregivers in scanning the codes to complete the online questionnaire.

### Outcomes

The primary outcome measure was the counselling quality rate. Following the UNICEF training framework, we defined high-quality counselling as achieving at least four out of six key counselling skills during the counselling session [14]. Furthermore, the average counselling duration was assessed in the two groups at both baseline and endline.

The secondary outcome measure was IYCF practices. We used indicators of complementary feeding practices recommended by the WHO/UNICEF in 2021 [18], including MDD, MMF, and MAD. These indicators were measured at both baseline and endline surveys. MDD indicates the minimum number of food groups (at least five out of eight) fed to children aged six to 23 months during the past 24 hours. MMF refers to the minimum number of times of solid, semi-solid or soft foods (but also including milk feeds for non-breastfed children) provided among children aged six to 23 months. MAD refers to children who had obtained both the minimum dietary diversity and minimum meal frequency for their age.

The third outcome measure was the frequency of consumption of seven food groups by children during the last month, with high intake defined as at least three times per week. Additionally, we compared differences in caregivers’ feeding knowledge and feeding practices between two age groups.

### Statistical analysis

We carried out statistical analysis with SAS, version 9.4 (SAS Institute Inc., Cary, North Carolina, USA). We used medians (MDs) and interquartile ranges (IQRs) to describe continuous variables, and numbers and proportions to describe categorical variables. To assess differences between the control and intervention groups, we conducted the Wilcoxon signed-rank test for continuous variables and a χ^2^ test for categorical variables. Moreover, we employed a difference-in-differences (DID) approach in a linear model to estimate the net effect of the intervention. The significance level was set at *P* < 0.05. Because counselling services were delivered individually by village health workers to caregivers, the primary outcomes were measured and analysed at the individual level.

### Qualitative phase

We conducted two focus group discussions and six semi-structured interviews with the intervention group in July 2025 to gain a deeper understanding of the intervention's implementation and the challenges encountered during programme delivery.

### Sampling

We used convenience sampling to recruit participants from the intervention areas. VHWs from different villages within an intervention township participated in one focus group discussion. County- and township-level MCH workers from different townships in the intervention group participated in another focus group discussion. In addition, six caregivers of children aged six to 23 months from an intervention township were recruited for semi-structured interviews to explore their experiences with the counselling services.

### Data collection

One researcher from the Capital Institute of Paediatrics and two programme managers from UNICEF China conducted focus group discussions and semi-structured interviews. The study team developed the focus group guides. Discussions with county- and township-level MCH workers were conducted at the Changyang County Maternal and Child Health Hospital, and discussions with VHWs were conducted at the township hospital. Caregivers were also invited to participate at the township hospital. Discussions and interviews were conducted in Mandarin, typically lasted around 30 minutes, and were digitally recorded with each participant's permission. Recordings were transcribed verbatim in Chinese by Feishu translation software. Then, the study team member who participated in the focus groups validated the transcripts.

### Analysis

We analysed qualitative data using a content analysis approach developed by Graneheim and Lundman [19]. Two researchers (WQ and MN) independently analysed the data. First, they familiarised themselves with the material by reading through the written text several times. Then, they use MAXQDA, version 22 (VERBI Software GmbH, Berlin, Germany) to manage and structure the coding process. The meaning units – words, phrases, or paragraphs pertinent to the study topic – were identified and then condensed and assigned relevant codes. As coding advanced, similar codes were systematically clustered into subcategories through ongoing comparison. These subcategories were further examined, conceptually contrasted, and integrated into broader main categories. The two researchers compared the coding and discussed discrepancies until a consensus was reached. Representative quotations were selected to illustrate the main themes. Finally, the themes and selected quotations were translated into English by one researcher and reviewed by another to ensure accuracy. The qualitative findings were used to triangulate with the quantitative results, and perspectives from different participant groups were compared to strengthen the interpretation of the study findings.

## RESULTS

### Quantitative phase

#### Counselling quality assessment

The baseline and endline counselling quality assessments were conducted among 35 VHWs in the intervention groups and 36 VHWs in the control groups, with no significant differences in VHW characteristics between the two groups (Table S1 in the **Online Supplementary Document**). All VHWs were female, and the age was MD = 40 years. In both groups, more than 70% of VHWs completed junior college or university. In their daily work, the VHWs primarily engaged in newborn and postpartum home visits and the distribution of YingYangBao.

A total of 175 and 153 counselling sessions were assessed in the intervention township in September–October 2023 and June 2024, respectively, and 153 and 183 counselling sessions in the control township, respectively. At the baseline, the counselling quality rate was low in both groups (12.0% for the intervention group *vs*. 3.9% for the control group, *P* = 0.0072), while the rate increased to 84.8% in the intervention group at the endline, which was much higher than that in the control group (32.2%) (**Table 1**). The net effects for the counselling quality rate were significant between the groups (*P* < 0.0001). The overall counselling skills score between the two groups showed similar trends. The median scores of overall counselling skills for the intervention group increased from 2.50 at the baseline to 5.75 at the endline assessment, while the median scores for the control group increased from 1.66 at the baseline to 2.84 at the endline assessment, and there was a significant net effect between the two groups (*P* < 0.0001). The net effects for each skill were also significant between the groups. Furthermore, the median counselling duration increased to seven minutes (IQR = 5–9) in the intervention group at the endline, while the control group maintained a median duration of four minutes, consistent with its baseline level.

**Table 1:** Comparison of IYCF counselling quality assessment between the intervention and control groups*

Items	Baseline (n = 330)			Endline (n = 336)			DID *P*-value
	**Intervention group (n = 175)**	**Control group (n = 155)**	***P*-value**	**Intervention group (n = 153)**	**Control group (n = 183)**	***P*-value**	
Counselling quality rate, n (%)	21 (12.0)	6 (3.9)	0.0072	145 (84.8)	59 (32.2)	<0.0001	<0.0001
Overall counselling skills score	2.50 (1.49–3.28)	1.66 (1.25–2.48)	<0.0001	5.75 (5.32–6.00)	2.84 (1.58–4.25)	<0.0001	<0.0001
Obtaining the correct infant age	1.00 (1.00–1.00)	1.00 (1.00–1.00)	1.00 (1.00–1.00)	1.00 (1.00–1.00)	1.00 (1.00–1.00)	<0.0001	0.0129
Assessing infant feeding score	0.33 (0.12–0.58)	0.12 (0.04–0.54)	<0.0001	0.95 (0.70–1.00)	0.37 (0.29–0.62)	<0.0001	<0.0001
Analysing the feeding problems score	0.33 (0.00–0.670)	0.00 (0.00–0.33)	0.0004	1.00 (1.00–1.00)	0.65 (1.00–0.00)	<0.0001	<0.0001
Providing actionable advice to caregivers scores	0.40 (0.00–0.80)	0.00 (0.00–0.40)	<0.0001	1.00 (1.00–1.00)	0.4 (0.00–1.00)	<0.0001	<0.0001
Appropriately using the counselling card score	0.00 (0.00–0.00)	0.00 (0.00–0.00)	1.0000	1.00 (0.60–1.00)	0.00 (0.00–0.00)	<0.0001	<0.0001
Communication skills during counselling sessions score	0.50 (0.50–0.75)	0.50 (0.25–0.50)	<0.0001	1.00 (1.00–1.00)	0.50 (0.25–1.00)	<0.0001	<0.0001
Average counselling duration	4.00 (3.00–7.00)	4.00 (3.00–5.00)	0.0003	7.00 (5.00–9.00)	4.00 (3.00–7.00)	<0.0001	0.4465

DID – difference-in-difference, IQR – interquartile range, MD – median

*Presented as MD (IQR) unless specified otherwise.

#### IYCF practices evaluation

The IYCF practices evaluation was conducted among caregivers of children aged six to 23 months, with 159 participants in the intervention group surveyed in January 2025 (baseline) and 161 in August 2025 (endline); in the control group, 174 caregivers were surveyed at baseline and 165 at endline. There were no statistically significant differences in the characteristics of the surveyed children and their main caregivers between the intervention and control groups at either baseline or endline (**Table 2**). Notably, over 70% of main caregivers in both groups were mothers, and more than 60% had attended senior high school or higher education.

**Table 2:** Characteristics of surveyed children and their primary caregivers at the baseline and endline surveys*

Characteristics	Baseline (n = 333)			Endline (n = 326)		
	**Intervention group (n = 159)**	**Control group (n = 174)**	***P*-value**	**Intervention group (n = 161)**	**Control group (n = 165)**	***P*-value**
**Child**						
Gender			0.1664			0.8986
*Male*	87 (54.7)	82 (47.1)		82 (54.0)	88 (53.3)	
*Female*	72 (45.3)	92 (52.9)		74 (46.0)	77(46.7)	
Age group in months			0.2963			0.1350
*6–11*	57 (35.8)	53 (30.5)		61 (37.9)	100 (62.1)	
*12–23*	102 (64.2)	121 (69.5)		79 (46.1)	89 (53.9)	
**Main caregivers**						
Relationships			0.5923			0.0788
*Mother*	123 (77.4)	146 (83.9)		133 (82.6)	139 (84.2)	
*Father*	16 (10.1)	13 (7.5)		10 (6.2)	17 (10.3)	
*Grandparents*	19 (11.9)	14 (8.0)		17 (10.6)	8 (4.9)	
*Other*	1 (0.6)	1 (0.6)		1 (0.6)	1 (0.6)	
Age in years, MD (IQR)	34 (29–38)	32 (29–36)	0.2656	33 (30–38)	33 (30–38)	0.6012
Education			0.0758			0.6749
*Primary school or below*	7 (4.4)	7 (4.0)		7 (4.4)	9 (5.5)	
*Middle school*	42 (26.4)	39 (22.4)		40 (24.8)	46 (27.9)	
*Senior high school*	70 (44.0)	61 (35.1)		65 (40.4)	56 (33.9)	
*College or above*	40 (25.2)	67 (38.5)		49 (30.4)	54 (32.7)	

IQR – interquartile range, MD – median

*Presented as n (%) unless specified otherwise.

At baseline, no statistically significant differences were observed in either children’s feeding practices or caregivers’ feeding knowledge between the two groups (*P* > 0.05), indicating adequate comparability (**Table 3**). At the endline, however, significant differences were observed in MDD and MAD. The intervention group had a higher prevalence of MDD (70.2% *vs*. 59.4%, *P* = 0.0415) and MAD (58.4% *vs*. 46.7%, *P* = 0.0342) than the control group. DID analysis revealed a significant net intervention effect on MDD between the groups (*P* = 0.0367). Further analysis showed that the prevalence of MDD in the control group decreased significantly from 75.9% at baseline to 59.4% at endline (*P* = 0.0012), whereas the intervention group maintained an MDD prevalence of approximately 70% across both survey time points.

**Table 3:** Comparison of children’s feeding practices and caregivers’ feeding knowledge between the intervention group and control group at the baseline and endline surveys, n (%)

Items	Baseline (n = 333)			Endline (n = 326)			DID *P*-value
	**Intervention group (n = 159)**	**Control group (n = 174)**	***P*-value**	**Intervention group (n = 161)**	**Control group (n = 165)**	***P*-value**	
Infant and young child feeding practices							
Introduction of solid, semi-solid or soft foods 6–8 mo*	25 (83.3)	15 (75.0)	0.4705	23 (85.2)	26 (74.3)	0.2959	0.8701
*Minimum dietary diversity*	114 (71.7)	132(75.9)	0.3876	113 (70.2)	98 (59.4)	0.0415	0.0367
*Minimum meal frequency*	125 (78.6)	127 (73.0)	0.2318	120(74.5)	112 (67.9)	0.1848	0.8813
*Minimum acceptable diet*	98 (61.6)	102 (58.6)	0.5748	94 (58.4)	77 (46.7)	0.0342	0.2591
*Consumption of iron-rich or iron-fortified foods*	137 (86.2)	152 (87.4)	0.7482	142 (88.2)	148 (89.7)	0.6661	0.9523
*Continued breastfeeding at 12–23 mo*†	22 (21.6)	15 (12.5)	0.0708	12 (12.1)	14 (15.7)	0.4742	0.0744
Children’s food frequency for the last month							
*Grains, roots, tubers and plantains*	84 (52.8)	89 (51.2)	0.7591	90 (55.9)	66 (40.0)	0.0041	0.0674
*Vitamin-A-rich fruits and vegetables*	95 (59.8)	99 (56.9)	0.5981	108 (67.1)	69 (41.8)	<0.0001	0.0034
*Other fruits and vegetables*	88 (55.4)	97 (55.8)	0.9413	103 (64.0)	84 (50.9)	0.0171	0.0815
*Flesh foods*	71 (44.7)	74 (42.5)	0.6960	77 (47.8)	49 (29.7)	0.0008	0.0361
*Eggs*	77 (48.4)	87 (50.0)	0.7744	84 (52.2)	61 (37.0)	0.0057	0.0308
*Pulses (beans, peas, lentils), nuts and seeds*	37 (23.3)	50 (28.7)	0.2568	51 (31.7)	28 (17.0)	0.0019	0.0028
*Dairy products (yoghurts or cheese)*	27 (17.0)	28 (16.1)	0.8272	30 (18.6)	18 (10.9)	0.0491	0.2277
Caregivers’ feeding knowledge							
*Exclusive breastfeeding up to 6 mo*	71 (44.7)	75 (43.1)	0.7758	93 (57.8)	70 (42.4)	0.0056	0.0754
*Starting complementary food at 6 mo*	103 (64.8)	107 (61.5)	0.5349	106 (65.8)	101 (61.2)	0.3857	0.8588
*Continued breastfeeding for 2 y or beyond*	27 (17.0)	19 (10.9)	0.1093	48 (29.8)	7 (4.2)	<0.0001	0.0004

DID – difference-in-difference

*Denominator for this indicator is children aged 6–8 months.

†Denominator for this indicator is children aged 12–23 months.

For the frequency of high consumption of the seven food groups during the last month, children in the intervention group had higher proportions than those in the control group at the endline (*P* < 0.05). Additionally, significant net intervention effects were observed for four specific food groups: vitamin A-rich fruits and vegetables (*P* = 0.0034), flesh foods (*P* = 0.0361), eggs (*P* = 0.0308), and pulses (*P* = 0.0028). Moreover, more caregivers in the intervention group knew that children should be continued breastfed up to two years or above compared to those in the control group at the endline (29.8% *vs*. 4.2%), with a significant net intervention effect (*P* = 0.004).

### Qualitative phase

We recruited a total of 23 participants for the qualitative phase of the research, including eight VHWs, six caregivers with children aged six to 23 months, six township-level and three county-level MCH workers who provided supportive supervision. The inductive content analysis returned four categories: changes among VHWs; changes among caregivers; existing problems and obstacles; and project aid-tool (Table S2 in the **Online Supplementary Document**).

#### Changes among VHWs

In focus groups, the VHWs reported that their IYCF knowledge and service delivery capacity improved after training, with increased self-confidence in communicating feeding information, and that they gradually established an authoritative image among caregivers:

*From just having a rough idea at first, you know, to now being in a pretty professional field of expertise.* – VHW, focus group 1.

*Before, I only had a partial understanding, but now I'm better able to apply what I've learned to my work and it’s really made a big difference.* – VHW, focus group 1.

*At least for us personally, our skills have really improved. I mean, we do feel more authoritative now. Before, we were just reciting from the book, and we didn't dare to speak too theoretically with people. Now it's much better.* – VHW, focus group 1.

*Whether we're talking to the mothers or to the grandparents, we just feel more confident now.* – VHW, focus group 1.

In Changyang County, the community health workers – who also served as women's directors at the village level – had built deep trust with caregivers through their longstanding involvement in village public health work. This included regular activities such as folic acid and Ying Yang Bao distribution, as well as postpartum visits, which brought them into frequent contact with caregivers and children. This trust provided a strong foundation for the acceptance and effectiveness of their counselling.

#### Changes among caregivers

Caregivers reported improvements in both their feeding knowledge and practices. In terms of knowledge, they gained a clearer understanding of key IYCF principles, such as the appropriate timing for introducing complementary food and the importance of dietary diversity. Practically, they made improvements to complementary food diversity, frequency, and texture, were willing to try new nutrient-dense foods or meats, prepared separate meals tailored to their children's needs, and utilised feeding tools like blenders and high chairs. Additionally, caregivers noted that the counselling effectively addressed their feeding-related concerns, and they expressed high satisfaction with the services provided:

*At the beginning, my child’s diet was quite limited. If he didn’t like a food, we usually wouldn’t try again. After the counselling, we started to try new foods more often. Now he can eat almost everything, and we prepare a wider variety of foods for him.* – Caregiver 2, interview.

*We used to just hold the baby while feeding without paying much attention. After the counselling, we bought a baby chair so the child can sit and eat, and sometimes we feed the baby while we are eating together.* – Caregiver 5, interview.

#### Existing problems and obstacles

However, the programme implementation encountered several challenges. For the VHWs, their non-medical backgrounds left them feeling lack of sufficient professional expertise. Although they could identify feeding-related issues, they were unable to explain them in depth or answer specific questions raised by caregivers. For caregivers, multiple barriers hindered the adoption of scientific feeding practices. Geographically, remote residential locations have limited access to regular counselling services. Demographically, the high mobility of rural children disrupted the continuity of service delivery, and frequent changes of caregivers made it difficult to maintain consistent feeding practices. Particularly as grandparents, who often take on caregiving roles, hold deep-rooted traditional feeding beliefs and tend to adhere to conventional methods that are hard to change. Additionally, caregivers were frequently occupied with farm work, leaving them little time to prepare the complementary foods for children:

*Village health workers can identify some feeding problems, but it is sometimes difficult for them to explain the issues in depth. They can usually provide a few basic suggestions.* – Township MCH worker, focus group 2.

*The person who feeds the child is often not fixed. Sometimes the child is cared for by grandparents or other relatives. Because different caregivers have different feeding ideas, it is difficult to maintain consistent feeding practices.* – Township MCH worker, focus group 2.

#### Project aid-tool

A key feature of the project laid in its well-designed counselling cards and health education leaflets. Especially, the health education leaflets boasted multiple strengths: the content was authoritative and aligned with national IYCF guidelines, the language was concise and easy to understand, and the information was highly targeted to the needs of rural caregivers. Notably, the leaflets also enhanced counselling efficiency by serving as a visual aid for VHWs to explain complex information and as a handy reference for caregivers to review after counselling sessions:

*It’s simpler and clearer-you can grasp the key points without spending too much time.* – CHW, focus group 1.

*I think it’s great! It's so easy to read at a glance, and the amounts and textures are all crystal clear.* – CHW, focus group 1.

## DISCUSSION

Results of this study demonstrated that the ‘2 + 1’ IYCF counselling training model significantly improved the quality of IYCF counselling delivered by VHWs in the intervention group, with a marked increase in the counselling quality rate from 12.0% at baseline to 84.77% at endline, which was significantly higher than that in the control group (32.2%). The intervention's positive impact on counselling quality translated into meaningful improvements in key IYCF practices, particularly in MDD and MAD. At the endline, the intervention group had higher MDD (70.2% *vs*. 59.4%) and MAD (58.4% *vs*. 46.7%) prevalence than the control group.

It is important to note that the baseline survey for IYCF practices was conducted in January 2025, coinciding with the Chinese New Year. During this traditional festival, Chinese families typically prepare a wide variety of foods for celebrations, which accounts for the high prevalence of MDD at baseline in both groups (>70%). In addition, the endline survey was conducted in August, when food availability and dietary patterns may differ due to seasonal variation. Therefore, seasonal factors may have influenced the observed levels of dietary diversity at both time points. Despite these potential seasonal influences, the DID analysis suggested a significant net intervention effect on MDD, primarily characterised by the intervention's role in stabilising MDD rates at approximately 70% over the study period in the intervention group, in contrast to the substantial decline in the control group (75.9%–59.4%). These findings indicate that the intervention played a protective role in maintaining IYCF practices over time.

Since 2009, China has implemented the national basic public health service (NBPHS) programme, which provides essential public health services to all residents, with a focus on children, pregnant women, the elderly, and patients with chronic diseases, thereby addressing the main health problems of urban and rural residents [20,21]. In rural areas, village doctors are responsible for delivering the NBPHS [20,21]. However, most village doctors are male, old, and overburdened with work, and thus are unwilling to provide maternal health services to women [22–24]. Against this backdrop, our study focused on village MCH workers as alternative providers of IYCF counselling in rural villages. Qualitative results indicate that all the VHWs in this study were village women's directors, who work with women of childbearing age in their villages, responsible for improving the quality of local maternal and child health care services [25]. Our findings demonstrate that these CHWs, despite lacking professional medical backgrounds, have great potential to conduct IYCF counselling after ‘2 + 1’ training in rural China.

Recent reviews indicate that the format, duration, frequency, and content of health worker training are key factors influencing counselling quality [26]. In this study, the ‘2 + 1’ training model represents a novel format tailored to VHWs in rural settings. The two-day online training course offers them greater time flexibility and geographic accessibility [27], while the subsequent one-day in-person training session reinforces and facilitates the application of knowledge and skills acquired through the online module. Furthermore, qualitative results in this study indicate the ‘2 + 1’ training also improved VHWs’ self-efficacy and self-confidence, which in turn motivated them to deliver IYCF counselling to caregivers of children.

In addition to training design, supportive supervision is a probable approach to improve quality as well [26]. Evidence from a rural Nepal study reinforced the value of supportive supervision in motivating frontline workers [28]. Consistent with this, our study incorporated two rounds of supportive supervision, in which county and township-level MCH workers observed VHWs’ counselling sessions and provided timely, constructive feedback to reinforce skills and improve performance. Moreover, supportive counselling tools constitute another critical component of the intervention, contributing to improved counselling quality. The well-designed health education leaflet presenting key feeding recommendations serves as a visual aid to help VHWs explain complex concepts clearly, and as a convenient reference for caregivers to review post-counselling. This not only enhanced counselling efficiency but also strengthened the consistency and accuracy of information dissemination, addressing a common barrier to effective community-based nutrition interventions.

The findings of this study align with and extend the feasibility findings reported in our previous pilot study in Qinghai Province [10], which demonstrated the ‘2 + 1’ training model’s high acceptability, feasibility, and low cost, and could achieve more than 90% training coverage in rural China. Our results substantiate that this scalable training approach is not only operationally feasible but also effective in behavioural changes among both health service providers and caregivers. Notably, as China has already scaled up the ‘2 + 1’ training model to 1212 counties across all 31 provinces, our study provides robust evidence to support the national programme’s effectiveness, filling a critical gap in understanding the training model’s real-world impact on key feeding practice outcomes.

Moreover, based on our findings, we recommend that the national programme incorporate routine IYCF counselling and supportive supervision into the NBPHS framework and adequately recognise and compensate VHWs’ labour contributions, thereby strengthening the initiative’s long-term sustainability. Future scale-up could involve a collaborative model that trains both village MCH workers and village doctors to deliver IYCF counselling as part of their regular responsibilities, with village doctors providing medical expertise. This integrated strategy would reduce the workload burden on overstretched village doctors and ensure the sustainable delivery of high-quality IYCF services in resource-limited rural settings.

Several limitations should be considered when interpreting the findings of this study. First, this study was conducted as a pragmatic, community-based intervention evaluation embedded within routine programme implementation, rather than as a formal cluster-randomised controlled trial. Although intervention areas were allocated at random, the allocation followed existing administrative structures, which limited baseline comparability between groups. Because clustering was not explicitly accounted for in the statistical analysis, standard errors may have been underestimated, leading to an overestimation of statistical significance. Therefore, the findings should be interpreted with caution. Second, the sampling strategy may have introduced potential selection bias. Counselling-quality observations were based on convenience sampling, and information on IYCF practices was collected via a WeChat-based questionnaire, which required caregivers to read Mandarin and use the WeChat platform. Consequently, caregivers who were more digitally literate or more engaged with health services may have been more likely to participate, while those from more disadvantaged or lower-literacy backgrounds may have been underrepresented. This may limit the generalisability of the findings to more remote or lower-resource rural settings. Third, infant feeding practices were assessed using repeated cross-sectional surveys rather than following the same caregivers over time. This design is commonly used in large-scale programme evaluations because it is operationally feasible and allows assessment of population-level changes after policy implementation. However, the DID analysis reflects changes at the population level rather than behavioural changes among the same individuals, which limits causal interpretation of the findings. In addition, the qualitative component of this study was exploratory and involved a relatively small convenience sample. Although the interviews provided contextual insights into caregivers’ experiences with the counselling intervention, the integration between qualitative and quantitative findings was limited. Future studies using more rigorous mixed-methods designs could further explore the mechanisms and contextual factors influencing intervention effectiveness. Finally, the study was conducted in a single rural county in China, which may limit the generalisability of the findings to other rural settings.

## CONCLUSIONS

Our findings support that the combined two-day online and one-day in-person IYCF counselling training effectively improved the quality of VHWs' feeding counselling skills. The training, together with programme support, also helped maintain IYCF practices, particularly food diversity, at the population level in rural China. Further studies are needed to assess the generalisability of the training model in other areas in China.

## Additional material


Online Supplementary Document
