# Supplementary material for: Effectiveness of a combined online and in-person training model in improving village health workers’ counselling quality and infant and young child feeding practices: a mixed-methods study in rural China
Source: J Glob Health. 2026 Jun 19;16:04177. doi: 10.7189/jogh.16.04177 (PMC13280864; doi:10.7189/jogh.16.04177)
Supplement: Online Supplementary Document [file jogh-16-04177-s001.pdf]

Supplement to: Wu Q, Zhu X, Wang X, Meng N, Li J, Peng A, Zhang Y, Chang S. Effectiveness of a combined online and in-person training model in improving village health workers' counselling quality and infant and young child feeding practices: a mixed-methods study in rural China.J Glob Health. 2026;16:04177

Table S1. Characteristics of community health workers (VHWs) assessed by counselling quality*

**Characteristics**	**Intervention group (n = 35)**	**Control group (n = 36)**	**Total (n = 71)**	***P-value***
Age in years, MD (IQR)	39 (36–44)	42 (38–45)	40 (38–44)	*0.2552*
Sex				
Female	35 (100.0)	36 (100.0)	71 (100.0)	
Education background				
Middle school	7 (20.0)	5 (13.9)	12 (16.9)	*0.4921*
High school	28 (80.0)	31 (86.1)	59 (86.1)	
Professional high school	6 (17.1)	3 (8.3)	9 (12.7)	*0.3669*
Technical secondary school	3 (8.6)	6 (16.7)	9 (12.7)	
Junior college or University	26 (74.3)	27 (75.0)	53 (74.6)	
Daily work				
Basic public health checkup for children	7 (20.0)	7 (19.4)	14 (19.7)	*0.9531*
Newborn home visit	26 (74.3)	29 (80.6)	55 (77.5)	*0.5273*
Postpartum home visit	28 (80.0)	31 (86.1)	59 (83.1)	*0.4921*
Distribute Yingyangbao	21 (60.0)	28 (77.8)	49 (69.0)	*0.1053*

*Presented as n (%) unless specified otherwise.

Table S2. Project Implementation: categories and subcategories

Main category (4)	Subcategory (23)
Changes among VHWs	Knowledge improved
	become more professional
	Enhanced service delivery capacity
	Increased confidence in communication
	Established deep trust with families
Changes among Caregivers	Knowledge improved
	feeding practice improved
	solved feeding problem
	Satisfied the counselling
Existing Problems and Obstacles	Caregivers and children：
	● Remote residence
	● High population mobility affecting service continuity
	● Frequent turnover of caregivers
	● grandparents take care of children
	● Deeply entrenched traditional beliefs
	● Busy for farm work
	VHWs:
	● Insufficient professional knowledge
	● Unable to explain identified feeding problems in depth
	● Unable to answer specific questions raised by caregivers
	● feel lacking enthusiasm and find the work troublesome
Project aid-tool	Clear at a glance
	concise and easy to understand

authoritative

enhanced counselling efficiency
